# Supplementary material for: Adjuvant Docetaxel in Node-Negative Breast Cancer Patients: A Randomized Trial of AGO-Breast Study Group, German Breast Group, and EORTC-Pathobiology Group
Source: Cancers (Basel). 2023 Mar 3;15(5):1580. doi: 10.3390/cancers15051580 (PMC10001055; doi:10.3390/cancers15051580)
Supplement: Supplementary file 1 [file cancers-15-01580-s001.zip › cancers-2225172-supplementary.pdf]

Supplement

Adjuvant docetaxel in node-negative breast cancer patients: a randomized trial of AGO-Breast Study Group, German Breast Group and EORTC-Pathobiology Group

Running Head

NNBC 3 – Europe. Adjuvant docetaxel in node-negative breast cancer patients

Christoph Thomssen[1]*, Martina Vetter[1], Eva J. Kantelhardt[1,2], Christoph Meisner[3,4], Marcus Schmidt[5], Pierre M. Martin[6+], Florian Clatot[7], Doris Augustin[8], Volker Hanf[9], Daniela Paepke[10], Wolfgang Meinerz[11], Gerald Hoffmann[12], Wolfgang Wiest[13], Fred CGJ Sweep[14], Manfred Schmitt[10+], Fritz Jänicke[15], Sibylle Loibl[16], Gunter von Minckwitz[16], Nadia Harbeck[17] on behalf of the NNBC 3-Europe Study Group

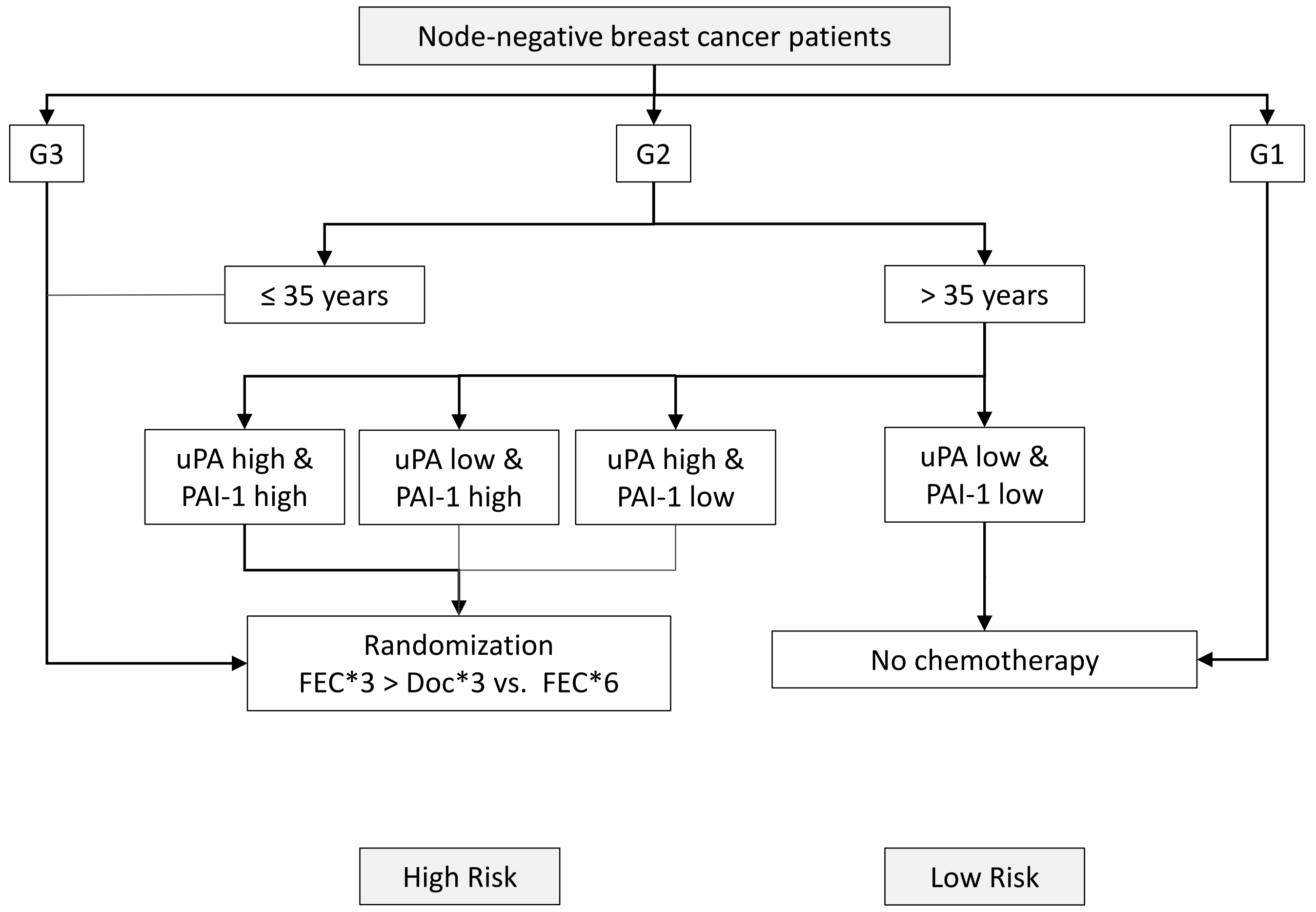

Figure S1: Tumor-biological risk assessment (using uPA/PAI-1)

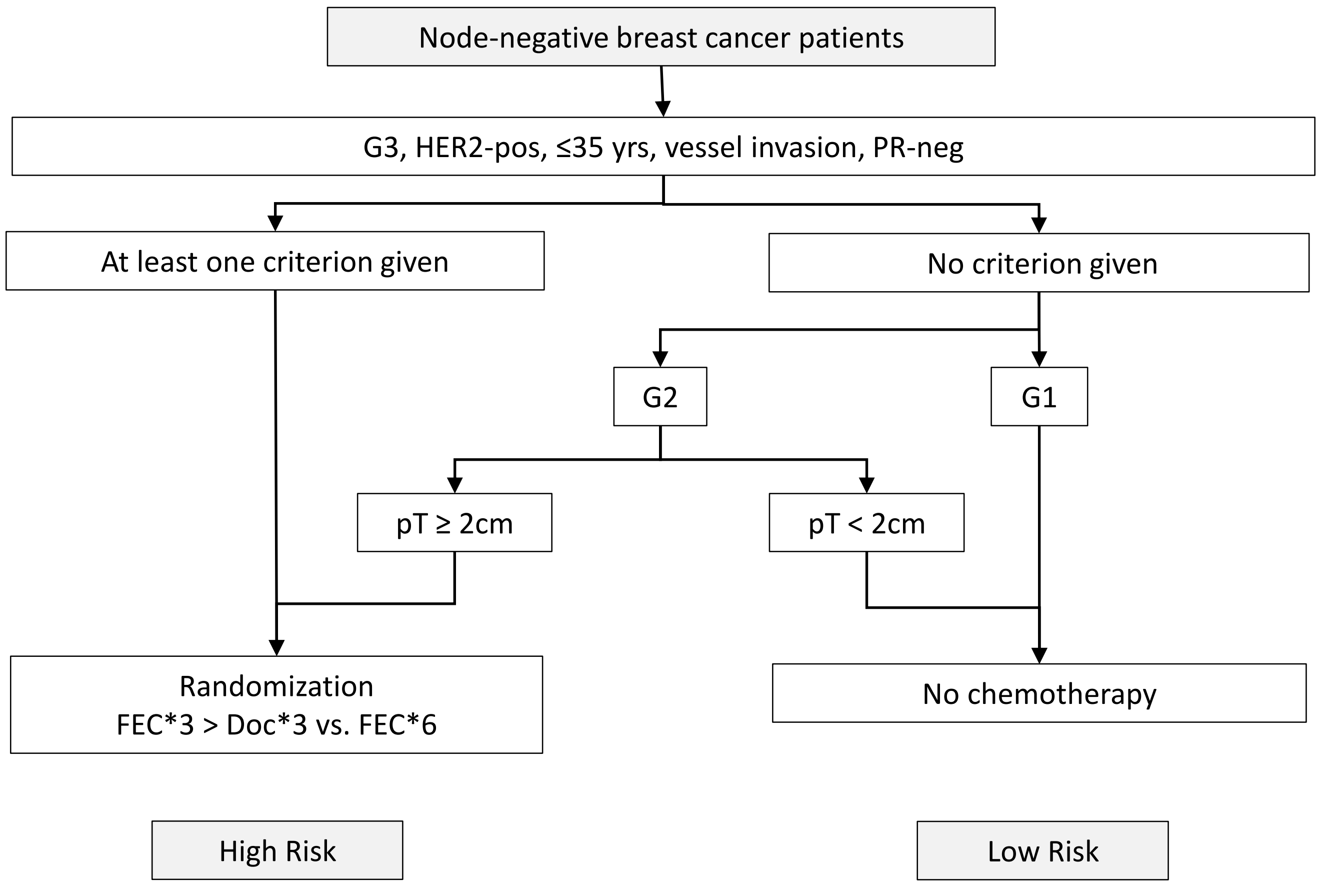

Figure S2: Clinico-pathological assessment

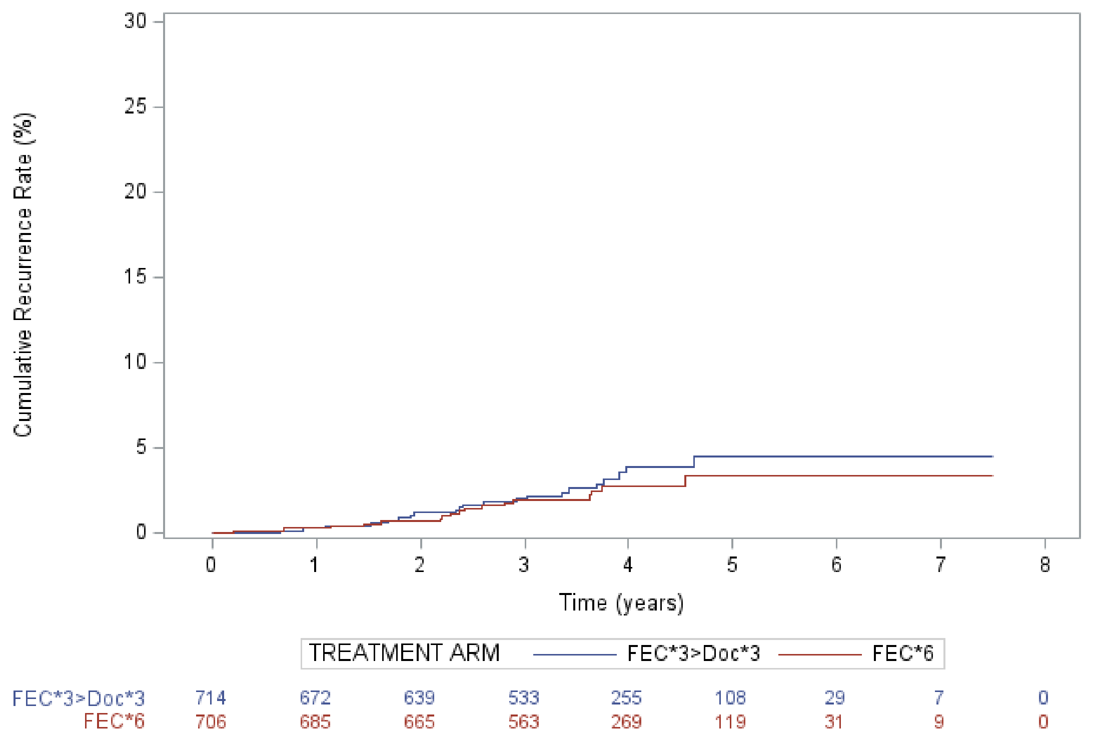

Figure S3: Survival estimates for RFI in luminal breast cancers stratified by FEC*3 > Doc*3 and FEC*6

The table presents the effective sample size for each interval (numbers at risk)

Table S1: List of the laboratories performing uPA/PAI-1 determination

**Laboratory**	**Determinations**	**Percent**
not documented	6	0,25%
Aachen RWTH	3	0,13%
Duisburg Bethesda Johanniter Klinikum	7	0,29%
Düsseldorf HHU Universitätsklinikum	27	1,13%
Wiesbaden HSK	1	0,04%
Halle (Saale) MLU UFK	725	30,44%
Hamburg UKE UFK	434	18,22%
Heidelberg Labor Limbach	132	5,54%
Augsburg Klinikum	6	0,25%
Mainz JGU UFK	355	14,90%
Marseille	295	12,38%
Montpellier	30	1,26%
Munich TUM UFK	349	14,65%
Mönchengladbach	1	0,04%
Bonn RFWU UFK	11	0,46%
**Total**	**2,382**	**100,00%**

Table S2: Severe chemotherapy toxicities (NCIC CTC grade 3 and 4) in relation (%) to the total number of chemotherapy courses

**Toxicity parameter**	**FEC*3 > Doc*3**		**FEC*6**	
Nausea	659	8.9%	893	12.3%
Arthralgia/myalgia	140	1.9%	24	0.3%
Vomiting	54	0.7%	78	1.1%
Infection	80	1.1%	48	0.6%
Stomatitis	51	0.7%	23	0.3%
Diarrhea	38	0.5%	15	0.2%
Headache	39	0.5%	13	0.2%
Allergic reaction	36	0.5%	5	0.1%
Sensory neuropathy	31	0.4%	9	0.1%
Alopecia	15	0.2%	13	0.2%
Skin/rash/desquamation	18	0.3%	4	<0.01%
Edema	7	0.1%	5	0.1%
Cardiac left ventricular function	5	0.1%	3	<0.01%
Dysuria	1	<0.1%	-	-
Fever	-	<0.1%	1	<0.1%
Death	1	<0.1%	-	-

Table S3: Number of events for each subgroup that has been calculated for the Forest plot calculation in Fig. 4

Variable	Subgroup	Events FEC	Events FEC-Doc
Age	≤ 50	34	29
	>50	27	37
Tumorsize	pT1	2	2
	pT2	59	63
Grading	G2	15	28
	G3	45	38
ER/PR	ER+ / PR+	15	22
	ER+ / PR-	7	10
	ER- / PR-	34	34
HER2-status	negative	47	47
	positive	14	18
Histology	ductal	55	53
	lobular	2	6
Local therapy	BCS	51	58
	MRM	10	7
Risk Assessment	tumor-biological	23	22
	clinico-pathological	38	44

Abbreviations: estrogen receptor status (ER), progesteron receptor status (PR), human epidermal growth factor receptor 2 (HER2), breast conserving therapy including radiotherapy (BCS), modified radical mastectomy (MRM)
